# Identification and quantification of virulence factors of enterotoxigenic *Escherichia coli* by high-resolution melting curve quantitative PCR

**DOI:** 10.1186/s12866-017-1023-5

**Published:** 2017-05-15

**Authors:** Weilan Wang, Ruurd T. Zijlstra, Michael G. Gänzle

**Affiliations:** 1 Department of Agricultural, Food and Nutritional Science, University of Alberta, 4-10 Ag/For Centre, Edmonton, AB T6G 2P5 Canada; 2 College of Bioengineering and Food Science, Hubei University of Technology, Wuhan, Hubei People’s Republic of China

**Keywords:** Virulence factors, ETEC, HRM-qPCR, Diarrhea, Fimbriae

## Abstract

**Background:**

Diagnosis of enterotoxigenic *E. coli* (ETEC) associated diarrhea is complicated by the diversity of *E.coli* virulence factors. This study developed a multiplex quantitative PCR assay based on high-resolution melting curves analysis (HRM-qPCR) to identify and quantify genes encoding five ETEC fimbriae related to diarrhea in swine, i.e. K99, F41, F18, F6 and K88.

**Methods:**

Five fimbriae expressed by ETEC were amplified in multiple HRM-qPCR reactions to allow simultaneous identification and quantification of five target genes. The assay was calibrated to allow quantification of the most abundant target gene, and validated by analysis of 30 samples obtained from piglets with diarrhea and healthy controls, and comparison to standard qPCR detection.

**Results:**

The five amplicons with melting temperatures (Tm) ranging from 74.7 ± 0.06 to 80.5 ± 0.15 °C were well-separated by HRM-qPCR. The area of amplicons under the melting peak correlated linearly to the proportion of the template in the calibration mixture if the proportion exceeded 4.8% (K88) or <1% (all other amplicons). The suitability of the method was evaluated using 30 samples from weaned pigs aged 6–7 weeks; 14 of these animals suffered from diarrhea in consequence of poor sanitary conditions. Genes encoding fimbriae and enterotoxins were quantified by HRM-qPCR and/or qPCR. The multiplex HRM-qPCR allowed accurate analysis when the total gene copy number of targets was more than 1 × 10^5^ / g wet feces and the HRM curves were able to simultaneously distinguish fimbriae genes in the fecal samples. The relative quantification of the most abundant F18 based on melting peak area was highly correlated (*P* < 0.001; r^2^ = 0.956) with that of individual qPCR result but the correlation for less abundant fimbriae was much lower.

**Conclusions:**

The multiplex HRM assay identifies ETEC virulence factors specifically and efficiently. It correctly indicated the predominant fimbriae type and additionally provides information of presence/ absence of other fimbriae types and it could find broad applications for pathogen diagnosis.

## Background

Post weaning diarrhea (PWD), especially enterotoxigenic *E. coli* (ETEC) related diarrhea, causes severe mortality and economical loss in the swine industry. Weaning imposes stress through the sudden change of diet and environment, and the interruption of immune protection from the sow’s milk. Taken together, these stressors increase the susceptibility of piglets to diarrhea [1]. A high proportion of poorly digestible dietary protein following weaning also favours colonization of the intestine with pathogens. Antibiotics, including prophylactic antibiotics [2, 3] and growth promoting antibiotics [1, 4] are used to maintain gut health. The increasing concerns of antibiotic resistance development resulted in a ban of antibiotics as growth promoters in several jurisdictions [5, 6], which makes control of post weaning diarrhea more difficult.

ETEC colonize the intestine by host-specific fimbriae that mediate adherence to receptors on the surface of the intestinal epithelium. After fimbriae-mediated colonization, ETEC strains produce toxins that disturb fluid homeostasis, thus causing severe diarrhea [1, 7–10]. CFA/I, CFA/II and E8775 fimbriae mediate the attachment of ETEC to the human intestinal epithelium [11, 12] whereas ETEC expressing K99 (F5), F41, F18, F6 (987P), and K88 (F4) fimbriae infect swine [9]. CFA/I fimbriae also act as a protective antigen which accelerate the immune response that protects the host from ETEC challenge [13]. Oral immunization with K88 fimbriae elicited a similar immune response as K88 fimbriae carrying ETEC infection in piglets; in both cases the immune response related to promotion of the gene expression of T cells producing IL-17 [14]. ETEC fimbriae thus play key roles in modulation of immune response which could supply more strategies for ETEC prevention by vaccines or receptor analogues [13–16]. Both heat-labile (LT) and heat-stable enterotoxins (STa and STb) are detected in the ETEC related diarrheal samples [17–19]; few strains additionally carry Stx2e [20]. The profile of virulence genes in swine isolates of ETEC varies with the age of the animals and the geographical location; ETEC carrying K88 fimbriae are more frequent in neonate animals while ETEC carrying F18 fimbriae are more frequent in weanling pigs [8–10, 19, 21]. Moreover, vaccination of piglets with a recombinant K88/LT vaccine provided superior protection against ETEC K88 challenge when compared to vaccine with K88 or LT antigens alone [22]. The diversity of virulence factors of ETEC and role of fimbriae as targets for therapeutic intervention highlight the need of effective methods that differentiate fimbriae of ETEC [18, 23–25].

PCR-based assays have become routine methods for rapid identification of .bacterial pathogens [23, 26–29]. High resolution melting (HRM) analysis is increasingly used to discriminate multiple targets in the same reaction (24, 30, 31). HRM analysis distinguishes single base changes in target sequences [32]. HRM-PCR assays were established as simple, fast, and accurate methods for rapid identification of lactobacilli [33]. Multiplex PCR-HRM also simultaneously distinguished diverse virulence factors of Shiga-toxin producing *E. coli* (STEC) [30]. In addition to the sensitivity, HRM analysis was suggested as cost efficient approach for differentiation of microorganism [31, 33, 34].

Quantitative HRM-PCR assays were first established to detect food adulteration, such as distinguishing admixtures to preparations of *Helleborus niger* for medical use [29] and detecting the presence of adulterations in basmati rice [35]. Relative quantification of template DNA with HMR-qPCR assays was initially based on the relationship between the level of normalised fluorescence at certain temperature and the proportion of the adulterant in the food or pharmaceutical preparation [27, 35–38]. A HRM-qPCR assay to simultaneously determine the relative proportions of four species of *Lactobacillus* in sourdough fermentation process achieved quantification by integration of the area of the melting peaks that are obtained by plotting the first derivative of the melting curve [39]. The peak area correlated linearly to the relative proportion of target sequences in a mixture of template DNA [39]. However, HRM-qPCR assays have not been developed for quantification of pathogens. This study therefore aimed to develop quantitative HRM methods to simultaneously identify and quantify five fimbriae types related to ETEC in swine, and to verify the reliability of the method in fecal samples obtained from weaned piglets.

## Methods

### Microorganisms and growth conditions

Two K88 antigen positive ETEC strains, *E. coli* strains ECL13795 (O149; virotype STb: LT: EAST1: F4) and ECL13998 (O149; virotype STa: STb: LT: EAST1: F4: Paa) were supplied by *Escherichia coli* Laboratory (University of Montréal, QC, Canada) and incubated at 37 °C overnight on Minca agar. Strains served as positive controls for the detection of K88 fimbriae, heat-labile toxin (LT), and heat-stable toxin (STa and STb) genes in qPCR and HRM-qPCR assays.

### Synthesized sequences and primers for qPCR and multiplex HRM-qPCR analysis

Partial sequence of genes encoding the K99, F41, F18 and F6 fimbriae biosynthesis or subunit (Table 1) were synthesized by gBlocks® Gene fragment (Integrated DNA Technologies, San Diego, California, USA) for use as positive controls in HRM-qPCR assay. The sequences were obtained from GeneBank database (http://www.ncbi.nlm.nih.gov) (Table 2). The specificity of primers was confirmed by PCR reaction with positive controls and DNA isolated from feces samples. For use as standards in HRM-PCR analysis, positive controls were amplified by PCR with specific primers (Table 2) and purified by agarose gel electrophoresis. The concentration of the amplicons was determined by nanodrop 2000 spectrophotometer (Thermo Fisher Scientific, Waltham, Massachusetts, USA) at 260 nm and amplicons were diluted to 10^10^ gene copies / μL.

**Table 1:** Synthetic DNA probes as positive controls for K99, F41, F18, and F6 fimbriae gene sequences for qPCR and multiplex HRM-qPCR analysis

Target (gene bank accesson No)	Target gene	Sequence	Location	Size (bp)
K99 fimbriae (X05797.1)	*E. coli* genes fanA and fanB involved in biogenesis of K99 fimbriae	GCATAAAACTCTGGTTCTTCTTGGCTGTTTATTTTTTTTTTCTATATGTTCAGTGTGTTATTTATACTCTTCCCTTTATTTTTGTTTTTTTTATGCCATATAATTCAATCAGCAGAGATGATTGGGATCATAAAAATGTCACTTGAGGGTATATGCGATCTTTTAATAAAGATGAATACTTGTTCAGGGAGAAACTTGGTTATCTTGTGAAAGGAATGGTTAAAGCAAGGTGCTTCCAATTATTAGTGGAGTTATCAAGTATACGTAGTTCTAGGG	314–589	276
F41 fimbriae (X14354.1)	*E. coli* fimbriae F41a gene for F41 fimbriae subunit	ACAATTGGGATGACCTCAGTCACAGCAACTATACTTCTGCAAATAAGGCATCTTATCTCTCTTATGGATCTGGTGTTTCTGCAGGTAGTACTTTAGTTATGAATTTAAATAAGGATGTTGCGGGTCGACTTGAATGGGTGG	916–1056	141
F18 fimbriae (KM260195.1)	*E. coli* isolate HDG_U113 FedA precursor (fedA) gene	AACACAGGGGCAGGAGGTTAAGGCGTCGAATAGCACTGTAAGTTTCGATGCATCAAAAGCAACTACGGAAGGTTTCAAATTTACTGCTCAACTGAAAGGTGGTCAAACCCCGGGTGACTTCCAGGGGGCAGCGGCTTACGCGGTTACTTACAAG	357–510	154
F6 (987P) fimbriae (M35257.1)	*E. coli* fimbriae 987P subunit gene	ACTAAATATTTAGTTCCAGCCTCCAATGATACTAGTGCATCAGGAGTTGGCGTATACATTCAGGACAACAACGCCCAGGCTGTGGAAATTGGTACTGAAAAAACTGTACCTGTGGTATCAAATGGCGGATTAGCTCTTTCAGACCAAAGTATTCCACTGCAAGCATACATCGGAACCACCACAGGGAATCCTGA	574–767	194

**Table 2:** Primers used for qPCR and multiplex HRM-qPCR analysis

Target gene	Sequence (5′--3′) (name)	Size (bp)	T_A_(°C)^a^	Reference
K99 fimbriae (*fan A*)	CACTTGAGGGTATATGCGATCTT (K99 F)	92	62	This study
	GACCTCAGTCACAGCAACTATAC (K99 R)			
F41 fimbriae Sub-unit A	GACCTCAGTCACAGCAACTATAC (F41 F)	110	62	This study
	CGACCCGCAACATCCTTATT (F41 R)			
F18 fimbriae (*Fed A*)	GGAGGTTAAGGCGTCGAATAG (F18 F)	90	62	This study
	CCACCTTTCAGTTGAGCAGTA (F18 R)			
F6 fimbriae (*Fas A*)	GTTCCAGCCTCCAATGATACT (F6 F)	128	62	This study
	GAAAGAGCTAATCCGCCATTTG (F6 R)			
K88 fimbriae (*fae G*)	GCACATGCCTGGATGACTGGTG (K88 F)	439	63	[54, 55]
	CGTCCGCAGAAGTAACCCCACCT (K88 R)			
*E. coli* (Universal stress protein A)	CCGATACGCTGCCAATCAGT (UspA F)	884	66	[26, 54]
	ACGCAGACCGTAGGCCAGAT (UspA R)			
Heat-labile toxin	CCGTGCTGACTCTAGACCCCCA (LT F)	480	68	[54, 56]
	CCTGCTAATCTGTAACCATCCTCTGC (LT R)			
Heat-stable toxins a	ATGAAAAAGCTAATGTTGGC (STa F)	193	65	[28, 54]
	TACAACAAAGTTCACAGCAG (STa R)			
Heat-stable toxins b	TGCCTATGCATCTACACAAT (STb F)	113	60	[54, 57]
	CTCCAGCAGTACCATCTCTA (STb R)			

^a^T_A_, primer annealing temperature

### Animals and growth environment

To validate the HRM-qPCR assay with animal samples, samples were collected at the Swine Research and Technology Centre (Edmonton, AB, Canada); animals were housed following the guidelines of the Canadian Council on Animal Care and trials were approved by the University of Alberta Animal Care and Use Committee. Samples from healthy animals were collected from crossbred weaned pigs (Duroc × Large White, aged 6–7 weeks) that were housed under standard sanitary conditions. Fecal samples were collected from 16 pigs housed in a clean room; 8 samples were collected from pigs with a fecal score of 5–6 at least once (diarrhea episode), and 8 samples were collected from pigs with a feces score of less than 5 (healthy control). Samples from animals with diarrhea were collected from crossbred weaned pigs (Duroc × Large White, aged 6–7 weeks) that were housed under poor sanitary conditions to induce diarrhea. Briefly, the housing was not cleaned before the piglets were moved to the facility and pooled feces from the sow herd were spread on the flooring twice: the day before new pigs were introduced and 1 week later. Feces was scored visually for consistency from 1 (solid feces) to 8 (watery diarrhea). Fecal samples were obtained from 14 pigs with a score 7 or greater for more than 3 three days.

### Sample collection and DNA extraction

Bacterial DNA was extracted from bacterial cultures with the DNeasy Blood & Tissue Kit (QIAGEN, Waltham, Massachusetts, USA). Fresh fecal samples were collected directly from rectum after stimulating animals to defecate. Fecal samples were placed in sterile plastic bags and stored at −80 °C. Frozen samples (0.2 g) were homogenized with ASL buffer and heated at 95 °C for 15 min to lyse cells and the supernatant was isolated by centrifuging at 18,800×g for 1 min. DNA extraction from feces samples followed the manufacturer instruction of QIAamp DNA stool minikit (QIAGEN). Template DNA was diluted to a concentration of 50 mg / L.

### Identification and quantification of ETEC fimbriae genes by individual / multiplex HRM-qPCR

All HRM-qPCR reactions were performed using a Rotor-Gene Q (QIAGEN) HRM-thermo cycler and Type-it HRM Kit (QIAGEN) Primers targeting five different porcine ETEC fimbriae genes (K99, F41, F18, F6 and K88) were designed with nearly identical annealing temperature (62 °C to 63 °C) (Table 2) to allow amplification in multiplex PCR reaction. HRM-qPCR reactions contained 12.5 μL 2× HRM Master Mix, 2 μL template DNA for individual reaction or 3 μL for multiplex reaction, 700 nM primers for individual reaction and 200 nM per target for multiplex detection to a final volume of 25 μL. The optimized PCR conditions were 5 min initial denaturation at 95 °C, 45 cycles of denaturation at 95 °C for 10s, annealing at 62 °C for 30s and extension at 72 °C for 25 s. During the HRM stage, temperature increased from 65 °C to 95 °C at the speed of 0.1 °C/ step and held for 2 s at each step.

qPCR reactions with single amplicons were calibrated by using serial 10-fold dilutions of positive controls to obtain standards containing 10^2^ to 10^8^ gene copies / μL as template. Multiplex HRM-qPCR combined absolute quantification of all template genes with relative quantification of the proportion of individual genes. A standard curve for the gene copy number of all target genes in multiplex amplification was established from serial 10-fold dilutions of positive controls to obtain standards containing 2 × 10^2^ to 2 × 10^8^ gene copies / μL of each of the five targets. The relative quantification was conducted on the basis of the linear correlation between the relative areas of the respective melting peaks to the relative proportion of specific target sequences in the mix of template DNA. The raw melting curve was deconvoluted by PeakFit software (Systat software Inc., San Jose, California, USA) using AutoFit Baseline and AutoFit Peaks I Residuals methods. To establish the calibration curves, five to seven different ratios of target were mixed with known concentration mixture DNA template and the ration of target to total DNA was plotted against the corresponding proportion of melting peak area to the total peak area. The calibration equations of the five fimbriae was verified by varying the percentage of the target fimbriae gene sequence in a template mixture containing the gene fragments of the four other fimbriae with identical gene copy number.

### Quantification of *uspA* and toxin genes by SYBR Green based qPCR analysis

Primers used for *uspA*, STa, STb and LT toxins quantification are listed in Table 2. qPCR was performed on a 7500 Fast real-time PCR system (Thermo Fisher Scientific) using MicroAmp Fast Optical 96-well reaction plate (Thermo Fisher Scientific). qPCR reactions contained 10 μL QuantiFast SYBR green master mix (Thermo Fisher Scientific), 2 μL (10 μM) primers, 1 μL template DNA, and water to a final volume of 20 μL. PCR conditions were as follows: initial denaturation 5 min at 95 °C, 40 cycles of denaturation at 95 °C for 30s, annealing at corresponding temperature (Table 2) for 30s and followed by 30 extension at 72 °C. At the melting stage, temperature increased with a speed of 0.5 °C/10s from 55 to 95 °C. To calibrate qPCR assays, target genes were amplified from chromosomal DNA of *E. coli* ECL13998, purified by agarose electrophoresis, and the concentration was determined by Nanodrop 2000 spectrophotometer (Thermo Fisher Scientific). The calibration of the qPCR assays was performed on the same instrument platform, with the same reagents and on the same day as the respective qPCR or HRM-qPCR assays.

## Statistical analysis

Experiments were conducted at least in triplicates and results were reported as means ± SEM. Data analysis was performed with Linear Regression model (PASW Statistics 18.0, Quarry Bay, HK, China) and *p* ≤ 0.05 was considered statistically significant.

## Results

### Identification of genes encoding ETEC fimbriae by multiplex HRM-qPCR

**Fig. 1:**
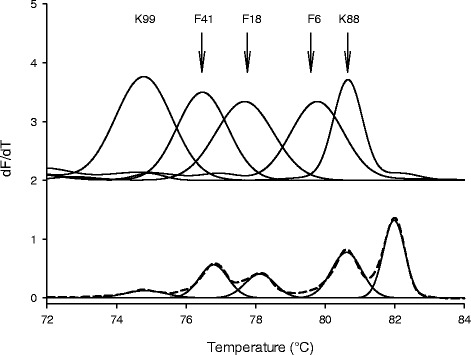
Melting curve of target sequence from positive controls by individual HRM-qPCR (*top*), melting curve of PCR products amplified from a mixed positive control including five different fimbriae gene sequence by multiplex HRM-qPCR (*bottom, dotted*) and the corresponding reprocessed melting curve by PeakFit software (*bottom, solid*)

A multiplex HRM-qPCR assay was developed for the simultaneous identification and quantification of five genes of fimbriae that mediate adhesion of ETEC to swine intestinal cells. HRM-qPCR separated the melting peaks of K99, F41, F18, F6 and K88 fimbriae gene amplicons with 1 to 2 °C difference (Fig. 1, Table 3). The melting curve results also indicated that melting temperatures of amplicons shift by up to 1 °C in multiplex PCR compared to the individual reactions. All melting temperatures obtained in multiplex assays were higher than that of individual amplicons; this difference was more pronounced for the genes encoding F6 and K88 fimbriae (Fig. 1). These results conform to the stabilizing effect of Evagreen, the fluorescent dye used in this HRM assay, on double stranded DNA. Tm values of dsDNA increased with the increasing dye concentration and decreasing amplicon concentration in most DNA binding dyes [40, 41]. The concentration of free Evagreen increases during melt curve analysis as the dye is released from double stranded DNA. During the melting curve stage of multiplex HRM-qPCR, dye release from the lower melting K99, F41 and F18 amplicons increased the dye concentrations and hence may have increased the binding strength and Tm shift for the higher melting F6 and K88 amplicons. With equal starting concentration of five targets sequences, amplified in multiplex conditions, the area of the five melting peaks differed substantially; the peak height of K99 was the lowest while K88 was the highest. As Evagreen showed equal preference for GC- or AT- rich amplicons, this results may indicate the lower affinity of Evagreen towards shorter double-strand DNA [42, 43]. The size of the amplicons ranged from 90 to 439 bp (Table 2). Due to the preferential binding to the dye, the peak area and height increased with increasing amplicons length in multiplex reaction (Fig. 1). The efficiencies of PCR with the 5 primer pairs did not differ substantially (Table 3), minimizing any additional effect of PCR efficiency on the area of the melting peaks.

**Table 3:** Melting temperature and calibration parameters for multiplex HRM-qPCR detection of K99, F41, F18, F6 and K88 fimbriae gene

	K99	F41	F18	F6		K88
Tm(°C)	74.7 ± 0.06	76.5 ± 0.12	77.6 ± 0.35	79.5 ± 0.78		80.5 ± 0.15
E ^a^	1.87	1.74	1.7	1.85		1.84
r_1_^2 b^	0.99	0.99	0.99	0.99		0.99
DNA range	1–20%	0.2%–16.7%	1%–100%	0.3%–4.8%	4.8%–60%	4.8%–20%
Slope ^c^	2.96	2.74	2.39	0.48	1.44	1.49
r_2_^2 d^	0.97	0.97	0.98	0.97	0.99	0.97

^a^primer efficiency determined by individual PCR

^b^r_1_^2^, correlation coefficient for standard curve determined by individual qPCR

^c^Slope for calibration curve correlating the area of the melting peaks area to the proportion of the template DNA

^d^r_2_^2^, correlation coefficient for standard curve correlating the area of the melting peaks to the proportion of the template DNA

### Quantification of genes encoding ETEC fimbriae by multiplex HRM-qPCR

**Fig. 2:**
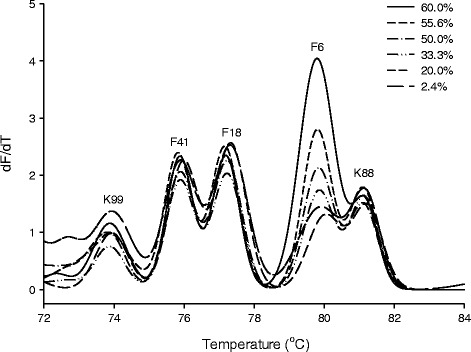
Calibration of F6 by changing the DNA range from 2.44 to 60% in the total gene copy number. Gene copy number of K99, F41, F18 and K88 was constant at 1× 10 ^10^. The percentages of F6 melting peak area and DNA range were plotted to establish the calibration equation parameters

To establish calibration curves for individual target genes in a mixture of all five genes, all five genes were mixed in equal molar concentrations and the proportion of one of the five was successively reduced. Template DNA containing fixed amounts of four target genes and a variable amount of one target gene were analysed by HRM-qPCR (Fig. 2). Calibration curves were established by correlating of the relative area of the melting peaks to the proportion of DNA in the mix of template DNA (Table 3). The calibration range was chosen to cover the content of target DNA in fecal samples (see below). The lowest detection limit was achieved for genes encoding F41 fimbriae, which were detected when their relative proportion exceeded 0.2% of total gene copy numbers. Two calibration curves were established for the gene encoding F6 fimbriae; one calibration curve covered the relative DNA content of 0.3 to 4.8%, a second calibration curve covered the relative DNA content of 4.8 to 60% (Fig. 2 and Table 3). The r^2^ of all regression equations was greater than 97% (Table 3).

### PCR quantification of genes encoding ETEC fimbriae in fecal samples

**Fig. 3:**
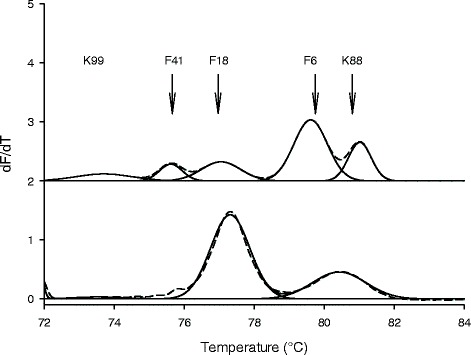
Melting curves of mixed positive control (*top, dotted*) and the same melting curve reprocessed by PeakFit software (*top, solid*), melting curve of a swine diarrhea feces sample (*bottom, dotted*) and the corresponding reprocessed melting curve (*bottom, solid*)

Fecal samples collected from 30 weaning pigs were analysed to assess the applicability of the multiplex HRM-qPCR methods. Samples were obtained from 14 weaning piglets where persistent diarrhea was induced by poor diet and poor sanitary conditions, and from 16 weaning pig that were kept in normal conditions and remained healthy (*n* = 8), or experienced diarrheal episodes (*n* = 8). ETEC fimbriae genes and toxins genes were quantified to determine if ETEC infection contributed to the persistent diarrhea or diarrheal episodes. HRM-qPCR distinguished amplicons of genes encoding ETEC fimbriae in fecal samples (Fig. 3). Multiplex HRM-qPCR analysis detected all five ETEC fimbriae in all fecal samples collected from animals with diarrhea (Table 3); moreover, the gene copy number of ETEC fimbriae types exceeded the detection limit in the most of fecal samples collected from healthy animals or animals with diarrhea episodes (Fig. 3, Table 4). The gene copy numbers of K99, F41 and K88 fimbriae were below the detection limit of the multiplex HRM-qPCR assay in several samples (Table 4). The area of melting peaks demonstrated that F18 was the predominant fimbriae type in animals with persistent diarrhea (Fig. 3, Table 4) but not in healthy animals. In animals with persistent diarrhea, high copy numbers of genes encoding fimbriae of ETEC were detected. Moreover, gene copy numbers of ETEC fimbriae did not differ from the gene copy number of *uspA*, which is present in all strains of *E. coli*, or the copy number of the gene encoding the STb toxin (Table 4). These results indicate that a majority of *E. coli* in the fecal samples were ETEC. However, in pigs with diarrheal episodes, or in healthy pigs, numbers of *E. coli* exceeded the numbers of ETEC more than 10,000-fold (Table 4).

**Table 4:** Gene copy number of ETEC fimbriae genes, total ETEC fimbriae, *Usp*A and toxins genes of diarrhea observed and health piglets detected by qPCR and HRM-qPCR methods. Data are presented as means of the log_10_ number of gene copies/g wet feces ± SEM in positive samples ^A^

Target genes	Diarrhea ^B^ Observations (*n* = 14)		Potential Diarrhea ^B^ Observations (*n* = 8)		Healthy animals (*n* = 8) ^B^	
	Individual qPCR	Multiplex HRM-qPCR	Individual qPCR	Multiplex HRM-qPCR	Individual qPCR	Multiplex HRM-qPCR
K99 fimbriae (*fan A*)*	5.85 ± 0.14 (14/14)^D^	7.52 ± 0.13 (14/14)	6.35 ± 0.04 (8/8)	3.99 ± 0.28 (6/8)	6.37 ± 0.05 (8/8)	3.97 ± 0.10 (5/8)
F41 fimbriae Subunit A*	6.67 ± 0.07 (14/14)	7.87 ± 0.13 (14/14)	4.46 + 0.07 (8/8)	3.62 ± 0.16 (6/8)	4.67 ± 0.14 (8/8)	4.47 ± 0.43 (7/8)
F18 fimbriae (*Fed* A)	8.51 ± 0.17 (14/14)	8.39 ± 0.22 (14/14)	5.03 ± 0.09 (8/8)	5.07 ± 0.08 (8/8)	4.89 ± 0.09 (8/8)	5.04 ± 0.14 (8/8)
F6 fimbriae (*F*as A)*	6.60 ± 0.06 (14/14)	7.81 ± 0.12 (14/14)	4.43 ± 0.10 (8/8)	3.90 ± 0.09 (8/8)	4.28 ± 0.10 (5/8)	4.25 ± 0.12 (7/8)
K88 fimbriae (*fae G*)	7.15 ± 0.07 (14/14)	7.51 ± 0.14 (14/14)	5.74 + 0.07 (8/8)	4.10 ± 0.23 (8/8)	5.52 ± 0.05 (8/8)	3.60 ± 0.18 (6/8)
ETEC ^C^	N/A	7.99 ± 0.17^a^ (14/14)	N/A	4.63 ± 0.06^b^ (8/8)	N/A	4.68 ± 0.13^b^ (8/8)
Universal stress protein A	9.21 ± 0.17 (14/14)	N/A	8.92 ± 0.20 (8/8)	N/A	8.80 ± 0.19 (8/8)	N/A
Heat-labile toxin	4.24 ± 0.23^a^ (9/14)	N/A	< 3^b^	N/A	< 3^b^	N/A
Heat-stable toxins a	6.22 ± 0.21^a^ (14/14)	N/A	5.11 ± 0.71^a^ (8/8)	N/A	3.02 ± 0.01^b^ (8/8)	N/A
Heat-stable toxins b	7.80 ± 0.18^a^ (14/14)	N/A	5.03 ± 0.09^b^ (8/8)	N/A	4.89 ± 0.09^b^ (8/8)	N/A

^A^the detection limit of individual qPCR was 10^4^ copies / g wet feces; the detection limit of multiplex HRM-qPCR was 10^5^ copies / g wet feces

^B^Diarrhea observation corresponds to fecal scores of 7 or higher for more than 3 days; potential diarrhea observation corresponds to fecal scores ranging from 5 to 6 at least once; healthy animals corresponds to fecal scores of less than 5;

^C^ETEC was calculated by the CT values and standard curve of HRM-qPCR detection for the feces samples, including the amplification of K99, F41, F18, F6, and K88. ETEC was calculated as the sum the gene copy numbers of all five fimbriae genes as obtained from the CT value of fecal samples by multiplex HRM-qPCR

^D^number of positive samples / number of total samples

N/A, not analysed

*means results detected by qPCR and HRM-qPCR are significantly different (*P* < 0.05)

Data in the same row (ETEC, Universal stress protein A, Heat-labile toxin, Heat-stable toxin a and Heat-stable toxin b) that do not share a common superscript are significantly different (*P* < 0.05)

### Comparison of multiplex HRM-qPCR to quantification with individual qPCR reactions

**Fig. 4:**
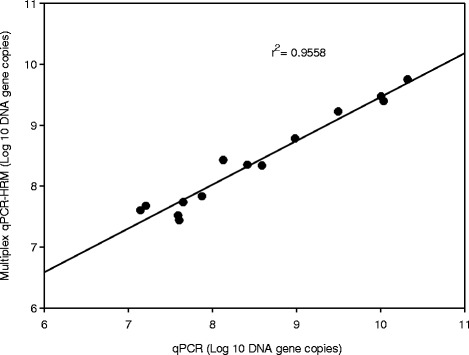
Scatter plot and regression analysis between the Log_10_ gene copy number of F18 fimbriae gene measured by qPCR and multiplex HRM-qPCR methods (*n* = 14)

To verify the reliability of multiplex HRM-qPCR, results of HRM-qPCR analysis were compared to specific qPCR assays as reference method for quantification of DNA. Multiplex HRM-qPCR identified fimbriae types without false positive results (Table 4). Multiplex HRM-qPCR detected the target DNA in fecal samples with a detection limit 10^5^ copies / g wet feces while the detection limit of qPCR assays ranged from 10^3^ to 10^4^ gene copies / g wet feces. The consistency of qPCR and multiplex HRM-qPCR quantification was analysed by regression analysis (Fig. 4). Correlation of data obtained for quantification of the most abundant F18 fimbriae revealed a r^2^ of 0.9558 (*P* < 0.001), demonstrating a high consistency between the two methods. However, the gene copy number obtained by multiplex HRM-qPCR analysis was inconsistent with the gene copy number obtained by qPCR for K99, F41 and F6 (*P* < 0.05). An r^2^ of 0.3572 was obtained when data for all five fimbriae were used for the correlation analysis. The difference between qPCR and multiplex HRM-qPCR analysis likely reflects overestimation of low abundance target genes by HRM-qPCR.

## Discussion

The diversity of virulence factors of swine ETEC and the occurrence of hybrid virotypes necessitates identification of pathogenicity for control and treatment of post weaning diarrhea in swine production [1]. F18 and K88 positive ETEC strains are the most widespread cause for the *E. coli* related post weaning diarrhea and edema disease in piglets [7, 44]. *E. coli* with F18 fimbriae cause diarrhea in weaned piglets. Neonatal pigs lack receptors for F18 fimbriae and ETEC with K88 fimbriae typically cause diarrhea in nursing piglets [1, 44]. More than 70% of F18 fimbriae positive ETEC strains isolated from diarrheal pigs in the US produce heat-stable enterotoxins (STa and STb). In contrast, strains expressing K88 fimbriae usually produce labile toxin (LT) and STb while STa production is infrequent [8, 45, 46]. Active immunization or passive immunization are used to protect neonatal and weaning pig since they are highly susceptible to ETEC infection [1]. Successful immunisation strategies target the different fimbriae addition to the toxins, and hence require knowledge on the fimbriae that are related to disease development [47]. Oral immunization with recombinant vaccines such as recombinant K88/LT vaccine [22] or recombinant K88/K99/F6/F41/F18 fimbriae proteins [15] was more advantageous in stimulation of systematic and mucosal immunity [22]. Strategies that employ receptor decoys to prevent binding of specific fimbriae types to the glycan receptor on the surface of the intestinal mucosa provide an alternative therapeutic option to prevent ETEC-induced diarrhea in young pigs. However, different fimbriae bind to different glycan receptors and hence require the use of different therapeutic oligosaccharides [48]. For example, EPS from *L. reuteri* was shown to prevent adhesion of K88 ETEC but its effect on ETEC expressing other fimbriae remains to be demonstrated [15]. In brief, several therapeutic options for diarrheal disease in piglets including specific recombinant vaccines target bacterial fimbriae and hence depend on diagnostic tools that identify the fimbriae type associated with the diarrheal pathogen. Because several ETEC strains expressing different fimbriae may be present simultaneously, diagnostic tools should be able to identify fimbriae types that are most abundant and hence most relevant for disease development.

HRM analysis discriminates sequence variations between amplicons by determination of high resolution melting curves with a precision of 0.1 °C [27]. Compared to the individual qPCR analyses, multiplex HMR-qPCR assays are a suitable, cost-effective and high-throughput strategy for qualitative or quantitative analysis of pathogenic *E. coli* [27]. Previous studies employed HRM-qPCR to confirm the presence of *E. coli* in an ETEC-challenged small intestinal segment perfusion model [16]. Differentiation between *E. coli* and other bacterial taxa was achieved by HRM analysis of amplicons of 16S rRNA genes; confirmation of strain identity was provided by qPCR analysis of strain-specific virulence factors [16]. Additionally, a multiplex HRM-PCR platform was established to discriminate among virotypes of *E. coli* on the basis of the presence or absence of 7 genes encoding virulence factors [49]. However, the choice of virulence factors that were included in the assay did not encompass those genes that are required to differentiate between different ETEC in swine. This study employed HRM-qPCR to differentiate between swine-associated ETEC strains expressing 5 different types of fimbriae. PCR primers were selected to obtain amplicons separated by 1–2 °C, which was sufficient to differentiate the genes in samples containing all five genes.

The multiplex HRM-qPCR assay established in this study not only identified genes encoding virulence factors of swine-associated ETEC, it also quantified their relative abundance. Most other quantitative multiplex HRM-qPCR methods achieve quantification of two or more amplicons based on the normalised fluorescence level [29, 36]. Quantification methods developed for basmati rice adulteration, however, allowed confident detection and quantification only when the percentage of adulteration was more than 15% [35–38]. Quantification of multiple amplicons based on the area under the melting peak was first developed to quantify *Lactobacillus* spp. in sourdough [39]. Robust identification and integration of melting peaks that are obtained by the df/dT derivative of the melting curves is achieved with standard chromatography software [39]. Reprocessing the melting curves with chromatography software also increased the accuracy of quantification by reducing the signal-to-noise ratio. The quantification method allowed accurate detection (R^2^ > 0.98) of multiple *Lactobacillus* species when the corresponding DNA content was more 0.2% of the total DNA [39]. This previous method used a single primer pair to obtain amplicons of 16S rRNA genes that differ in their melting temperature, therefore, concerns related to primer annealing and PCR efficiency of multiple primers for amplification of multiple genes were not addressed [39].

The multiplex HRM-qPCR developed in this study adopted the relative quantification method based on melt peak area [39] but applied five specific primer pairs rather than one universal primer pair. Primers were selected to obtain identical primer annealing temperatures but amplicons that differ in the melting temperature. Moreover, PCR conditions of multiplex amplification were optimised to achieve similar amplification efficiency. Because primer design for HRM-qPCR is constrained by the necessity of obtaining 5 primer pairs with the same annealing temperature but amplicons having different melting temperatures, the PCR efficiency was not further optimized. The method developed in this study detected genes encoding target fimbriae if their proportion of the total target DNA exceeded 0.2%. The relative quantification results of multiplex HRM-qPCR was comparable to individual qPCR for the predominant fimbriae type but HRM-qPCR provided a higher relative proportion for low abundance targets when compared to conventional qPCR. Although qPCR is considered the “gold standard” for sequence-specific quantification of DNA, discrepancy with other quantitative methods were also observed in comparison of qPCR to other methods for quantification of DNA, e.g. microarray analysis or high-throughput sequencing [50, 51]. Moreover, culture-based methods may be superior to DNA-based methods for quantification of viable *E. coli* in food and intestinal samples [52].

## Conclusion

As specific receptors on the host epithelia cells mediates the adhesion and colonization by ETEC, the susceptibility of swine to ETEC infection is determined by animal lineage and age [53]. Enterotoxins typically occur combined with specific serogroups and fimbriae [8]. The multiplex HRM-qPCR assay developed in this study distinguished five different fimbriae gene by optimizing the combination of primer pairs and reaction conditions. Moreover, the relative quantification based on melt curve area confirmed the prevalence of F18 in weaned pigs, and indicated that ETEC was associated with persistent diarrhea in weaning piglets. Accurate diagnosis of major fimbrial antigens and virulence determinants by multiplex HRM-qPCR may thus provide the basis for disease prevention [1], and to develop treatments targeting ETEC on the basis of their fimbriae type [54].
